# Cardiovascular Risk Reduction in Metabolic Dysfunction-Associated Steatotic Liver Disease and Metabolic Dysfunction-Associated Steatohepatitis

**DOI:** 10.1007/s11886-024-02185-5

**Published:** 2025-01-18

**Authors:** Johannes Bernhard, Lukas Galli, Walter S. Speidl, Konstantin A. Krychtiuk

**Affiliations:** Division of Cardiology, Department of Internal Medicine II, Medical University of Vienna, Waehringer Guertel 18-20, 1090 Vienna, Austria

**Keywords:** Metabolic dysfunction-associated steatotic liver disease, MASLD, Metabolic dysfunction-associated steatohepatitis, MASH, Statin, Obesity, Diabetes, Metabolic syndrome, Hypertension

## Abstract

**Purpose of Review:**

Metabolic dysfunction-associated steatotic liver disease (MASLD) is the most common chronic liver disease, characterized by hepatic steatosis with at least one cardiometabolic risk factor. Patients with MASLD are at increased risk for the occurrence of cardiovascular events. Within this review article, we aimed to provide an update on the pathophysiology of MASLD, its interplay with cardiovascular disease, and current treatment strategies.

**Recent Findings:**

Given their high burden of cardiovascular comorbidities, patients with MASLD or MASH should undergo regular cardiovascular risk assessment using established risk models. In the absence of liver-specific therapies, therapeutic strategies should focus on improving cardiometabolic risk factors. Patients require a multimodal and multi-stakeholder treatment approach, including optimization of lifestyle, dysglycemia, obesity, and dyslipidemia. Statin treatment represents a safe and effective but often underused therapy in the management of at-risk patients with MASLD and MASH. Novel promising approaches include the use of GLP-1 receptor agonists, especially in, but not limited to, patients with cardiovascular disease and obesity.

**Summary:**

Patients with MASLD and MASH are at high cardiovascular risk requiring a multi-modal therapeutic approach including regular cardiovascular risk assessment, as well as lifestyle and pharmacological interventions. Statin therapy represents an inexpensive, safe and effective therapy across the spectrum of non-alcohol related steatotic liver diseases without major safety concerns. More prospective, randomized trials in patients with MASLD and MASH are needed.

## Introduction

 Metabolic dysfunction-associated steatotic liver disease (MASLD), formerly known as non-alcoholic fatty liver disease [1], affects approximately 25–30% of the world population with a rapidly increasing global burden [2, 3]. MASLD is defined as evidence of hepatic steatosis in the presence of at least one (out of five) cardiometabolic risk factors without other causes of hepatic steatosis such as excessive alcohol intake or chronic viral or other forms of hepatitis present (Table 1). In the presence of both alcohol intake and cardiometabolic risk factors in a patient with steatotic liver disease, the disease is termed metabolic and alcohol related/associated liver disease (MetALD), acknowledging the continuum of disease contributing factors. MASLD may progress to metabolic dysfunction-associated steatohepatitis (MASH), a disease characterized by hepatocyte injury and inflammation and the development of liver fibrosis. This progression increases the risk of cirrhosis and hepatocellular carcinoma, both linked to high mortality.

**Table 1:** Diagnostic criteria for MASLD

Diagnosis	Diagnostic Criteria	
Metabolic Dysfunction Associated Steatotic Liver Disease (MASLD)	Hepatic Steatosis	Presence of at least one of the following five cardiometabolic risk factors and absence of other causes of liver steatosis
		(1) a body mass index (BMI) of 25 or above or a waist circumference of above 94 cm in men or 80 cm in women
		(2) fasting serum glucose of 100 mg/dL or above or a 2 h post-load glucose serum level of 140 mg/dL or above or HbA1c ≥ 5.7% or type 2 diabetes or treatment for type 2 diabetes
		(3) blood pressure of ≥ 130/85 mmHg or specific antihypertensive treatment
		(4) plasma triglycerides ≥ 150 mg/dL, or lipid lowering treatment
		(5) plasma high density lipoprotein-cholesterol (HDL-C) of ≤ 40 mg/dL in men or ≤ 50 mg/dL in women or lipid lowering treatment
Metabolic Dysfunction-Associated Steatohepatitis (MASH)	Evidence of Steatohepatitis	Presence of at least one of the following five cardiometabolic risk factors and absence of other causes of liver steatosis (see above)
Metabolic and Alcohol related/associated Liver Disease (MetALD)	Diagnostic Criteria of MASLD	Increased consumption of alcohol (140–350 g/week for females and 210–420 g/week for males)
Alcohol related Liver Disease (ALD)	Diagnostic Criteria of MASLD	Consumption of more than 350 g (females) or 420 g (males) of alcohol per week

However, in patients with MASLD, cardiovascular disease is the leading cause of mortality given the high burden of cardiovascular comorbidities [4, 5]. Within this review, we aim to provide an update on the new nomenclature of MASLD, review its pathophysiology and its close connection with cardiovascular disease, and highlight current treatment strategies. We focused on cardiovascular therapies such as statins, a safe and beneficial but often underused therapy. Throughout the review, we will adhere to the novel nomenclature, which underscores the importance of (cardio-)metabolic dysfunction in the pathogenesis of the disease spectrum.

## Pathophysiology of MASLD and Associated Cardiovascular Disease

MASLD represents the hepatic manifestation of metabolic syndrome and is closely connected to obesity, changes in hepatic insulin resistance, and glucose metabolism. It is primarily characterized by intrahepatic lipid accumulation [3], but the exact mechanisms leading to hepatic lipid accumulation and ultimately liver steatosis and dysfunction are incompletely understood [6]. Ultimately, an imbalance between lipid synthesis and absorption and hepatic liver elimination is the hallmark of MASLD pathogenesis [6, 7]. 

Sources of hepatic lipid intake include circulating free fatty acids, de novo generation of free fatty acids from nonlipid precursors and dietary intake. De novo lipogenesis from nonlipid precursors such as glucose and fructose [8] generates ectopic fatty tissue, which has been shown to lead to insulin resistance, followed by hyperinsulinemia [9]. Besides leading to gluconeogenesis and causing more insulin to be secreted, hyperinsulinemia is reinforced by lower rates of insulin clearing in MASLD [10]. Insulin resistance in turn, limits the ability of insulin to suppress lipolysis and store lipids, increasing the amount of circulating free fatty acids [11, 12]. Obesity is further often characterized by lower circulating levels of adiponectin, a hepatoprotective insulin-sensitizing adipokine with anti-inflammatory and anti-fibrotic effects [13], which has been described as a biomarker of MASLD disease severity [14]. 

Ongoing hepatic damage caused by the influx of free fatty acids, insulin resistance, and an overactive immune system ultimately propagates the development of steatohepatitis and hepatic fibrosis, a disease state now termed MASH [3]. Hepatocyte injury causes the release of damage-associated molecular patterns (DAMPs) which are recognized by Kupffer cells, the liver resident macrophages, via pattern recognition receptors such as toll-like receptors. Such activated Kupffer cells produce proinflammatory cytokines further exacerbating liver injury [15]. This in turn causes the release of growth factors, culminating in liver fibrosis and the clinical appearance of MASH [16].

The metabolic dysregulation seen in MASLD and MASH generates a pro-atherogenic milieu, including systemic inflammation, oxidative stress, insulin resistance, a pro-atherogenic lipid profile as well as endothelial dysfunction. Ectopic fatty tissue for example activates the inflammation cascade and causes oxidative stress, thereby directly influencing atherogenesis [9]. Alterations in the hepatic lipid metabolism in patients with MASLD were shown to result in atherogenic dyslipidemia, a pathologic lipid particle distribution characterized by elevated levels of plasma triglycerides and small-dense low-density cholesterol (LDL-C) lipoprotein particles and a decrease of high-density lipoprotein cholesterol (HDL-C) [17]. Beside well-known and extensively characterized atherogenic mechanisms, mechanisms specific to MASLD further exacerbate this process. Elevated levels of saturated fatty acids caused by an increase in hepatic de novo lipogenesis [9] for example have been shown to activate toll-like receptors (TLR) 2 and 4, which have previously been implicated in the initiation and progression of atherosclerosis [18–20]. 

## MASLD and Cardiovascular Disease

As the diagnosis of MASLD requires the presence of at least one cardiometabolic risk factor and as MASLD is the hepatic manifestation of metabolic syndrome, those two conditions have a significant overlap [2]. This close relationship is demonstrated in observational studies, demonstrating a higher CVD incidence [21], a stronger disease burden and a higher mortality [22] in patients with MASLD [23, 24]. While some analyses suggested that even after adjustment for conventional risk factors, MASLD remains an independent risk factor for the development of CVD [25, 26], in clinical practice, it has proven challenging to dissect these closely intertwined conditions and isolate MASLD as an independent risk factor of CVD. This has resulted in somewhat conflicting clinical practice guideline recommendations issued by scientific associations, with the American Heart Association (AHA) and the European Society of Cardiology (ESC) offering different opinions [27, 28]. Within the most recent *2021 ESC Guidelines on cardiovascular disease prevention in clinical practice*, the task force makes the argument that presence of MASLD does not increase CVD risk beyond traditional risk factors [28] but nevertheless suggests that such patients should have their CVD risk calculated and be screened for presence of diabetes. On the contrary, in a *Scientific Statement on Nonalcoholic Fatty Liver Disease and Cardiovascular Risk*, a joint author group from several councils of the AHA argues that MASLD is in fact an independent risk factor for atherosclerotic CVD [27, 29]. 

As the burden of CVD in patients with MASLD is evident, the most recent guidelines of the ESC, AHA, American Association for the Study of Liver Diseases (AASLD) and the European Association for the Study of the Liver (EASL) all recommend regular cardiovascular risk assessment and the need for more stringent assessment in patients with MASLD at high risk, such as in patients with type 2 diabetes mellitus [28, 30–32]. Although the two most frequently used prediction scores, the Framingham risk score and the atherosclerotic CVD risk score, have been validated in small cohorts for patients with MASLD [33, 34], the most recent guidelines do not recommend the use of any specific risk stratification tool. Yet another, novel, cardiovascular risk score is the SCORE-2 score promoted by the ESC [35], which has to date not been validated in patients with MASLD. Patients identified as having a high or very high cardiovascular risk require a multimodal and multi-stakeholder treatment approach, while statin treatment and lifestyle interventions should be initiated early in the disease trajectory.

Changes in lipid and glucose metabolism are assumed to be the main drivers of increased cardiovascular risk [36], therefore, therapeutic approaches are mainly focused on modification of such risk factors.

## Management of CVD Risk in MASLD and MASH

**Fig. 1:**
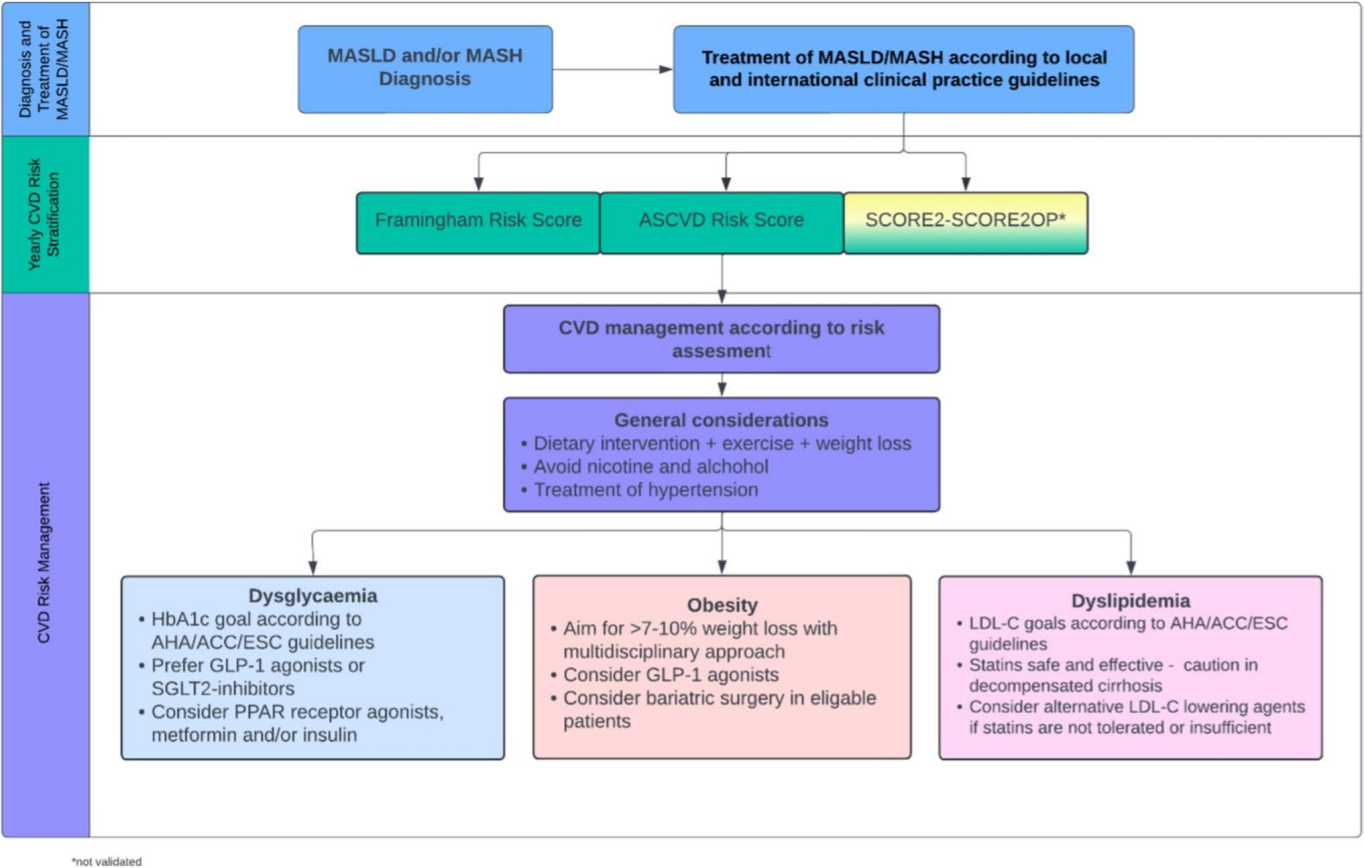
Diagnostic and therapeutic approach to patients with MASLD and MASH. Given their high burden of cardiovascular comorbidities, patients with MASLD and MASH should undergo regular cardiovascular risk stratification. Central to a multimodal treatment approach is lifestyle modification, including exercise, diet optimization, and weight loss. Depending on the estimated cardiovascular risk, initiation on specific therapies for the treatment of dyslipidemia, obesity, and dysglycemia is indicated. Key: MASLD: metabolic dysfunction-associated liver disease; MASH: metabolic dysfunction-associated steatohepatitis; CVD: cardiovascular disease; ASCVD: atherosclerotic cardiovascular disease; HbA1c: haemoglobin A1C, AHA: American Heart Association; ACC: American College of Cardiology; ESC: European Society of Cardiology; GLP-1: glucagon-like peptide-1; SGLT2: sodium-glucose cotransporter 2; PPAR: peroxisome proliferator–activated receptor; LDL-C: low-density lipoprotein cholesterol

The long-term goals in the treatment of patients with MASLD and MASH include improved survival, the prevention of diabetes mellitus, cardiovascular disease and associated events, the prevention of progression to liver cirrhosis and hepatocellular carcinoma as well as improvement in quality of life. Therefore, treatment should involve multidisciplinary care based on individualized plans tailored to the patient’s specific risk factors (Fig. 1).

### Lifestyle Modification

Lifestyle management represents a cornerstone in the treatment of MASLD and MASH [37]. The effects of diet, increased exercise, and subsequent weight loss not only positively impact cardiovascular health but also improve various parameters and risk factors associated with MASLD and MASH. While the effects of healthy diets such as the Mediterranean diet have been established [38], more recent data from a large meta-analysis points to the essential role of total caloric intake, showing significant improvements in liver function in patients with lower caloric intake [39]. 

Inadequate physical activity and a sedentary lifestyle were shown to be independent predictors of MASLD [40]. Recommendations on physical activity suggest 150 or more minutes of exercise weekly [31, 32], primarily stemming from studies demonstrating positive effects in preventing and resolving fatty liver disease, with improvements being independent of changes in body mass index (BMI) [41, 42]. Sufficient weight loss has also been shown to positively impact markers of liver damage and other MASLD biomarkers, with more pronounced changes seen in individuals with greater reduction [43]. Prospective data indicates positive effects on liver fat in patients with > 5% weight loss, with patients > 7–10% showing an additional reduction in inflammatory markers and fibrosis [44]. These findings are reflected in the EASL and AASLD clinical practice guidelines, with emphasis given to the need for a multidisciplinary approach, as achieving and sustaining weight loss presents significant challenges for the majority of patients [31, 32]. 

### Statin Treatment

Statins, or HMG-CoA reductase inhibitors, are recommended as the first line treatment of hyperlipidemia in patients with established CVD as well as in individuals at high risk for cardiovascular diseases including type 2 diabetes, obesity and metabolic syndrome by current clinical practice guidelines [30, 45, 46]. Patients with MASLD are oftentimes characterized by an atherogenic lipid profile or other cardiometabolic risk factors requiring statin therapy and are at elevated risk of CVD [47, 48]. Therefore, moderate to high intensity statin therapy depending on CVD risk is recommended for CVD risk reduction in MASLD patients across the spectrum of the disease and the severity of liver damage [31]. However, prospective, randomized data regarding the effects of statin therapy on cardiovascular disease endpoints is scarce and no randomized controlled trials have evaluated effects of statins on liver histological endpoints.

In clinical practice however, statins are dramatically underused in patients with MASLD and dyslipidemia [49–51]. Several analyses within various healthcare systems across the US and Europe showed that nearly half of all patients are not on a statin despite indicated by clinical practice guidelines. Even more dramatically, in one analysis, nearly a third of patients with at least one indication for statin treatment and concomitant MASLD were not receiving statin treatment. Predictors of statin underuse were provider awareness of steatosis, elevated transaminases or presence of more advanced disease stages, likely reflecting fear of liver-related side effects [49, 50]. Oftentimes statin treatment is discontinued in the presence of elevated transaminases for the fear of liver-related drug injury. Elevated transaminases in the setting of MASLD or MASH however are most often related to the liver disease itself which is caused and fueled by cardiometabolic risk factors that are targeted by statin treatment, thus representing a therapeutic dilemma in clinical practice [52]. 

Importantly, in a post-hoc analysis of the GREACE study including patients with abnormal liver tests at baseline, likely caused by MASLD, those patients treated with a statin actually showed improvement in their liver tests and a greater reduction in cardiovascular events. Furthermore, the benefit of statin therapy on cardiovascular outcomes was shown to be more pronounced in this patient collective than in patients without elevated liver enzymes and less than 1% of participants discontinued statin treatment based on transaminase levels higher than three-times the upper limit of normal [53]. Even in patients with established liver cirrhosis stage A or B within the Veterans Health Administration database, statin exposure was associated with a significant decrease of mortality and was considered safe [54]. 

Despite the hesitation by some clinicians to prescribe statins with established liver disease [55], the safety profile of statin therapy has been thoroughly established in patients with diagnosed MASLD and MASH [56]. A large meta-analysis of 13 studies could demonstrate that liver function tests and liver histology were actually improved in patients treated with statins with no significant changes in fibrosis stage [57]. Although current guidelines emphasize that statin therapy is contraindicated in patients with decompensated liver cirrhosis or acute liver failure, the use of statins in patients with other chronic liver disease is considered safe and is clearly indicated in patients with MASLD and MASH with manifest dyslipidemia with LDL-C levels above target range [31, 32, 56]. 

Not only are statins safe in patients with liver disease, observational studies also suggest positive results regarding liver related endpoints, and data from a meta-analysis point to lower risk of fibrosis and MASH in patients treated with statins [58]. Beneficial effects of statin treatment were also demonstrated in patients with advanced fibrosis and type II diabetes [59] and obese individuals, which reduced risk of non-alcoholic fatty liver in obese individuals [60]. 

Despite the relatively low incidence of hepatocellular carcinoma (HCC) associated with MASLD compared to HCC attributable to other etiologies such as hepatitis C virus (HCV) infection, the prevalence of HCC remains significant [61]. Although small prospective studies are currently still ongoing [62, 63], in a large meta-analysis of 24 studies with over 2 million patients included, statin use was associated with a 46% reduced incidence of HCC in comparison to non-statin users, an effect which may be attributable to the anti-inflammatory properties statins have demonstrated [64]. 

In summary, evidence from smaller observational studies and meta-analyses point to potential beneficial effects of statin treatment in the MASLD/MASH patient cohort. However, there is a lack of prospective, adequately sized, randomized controlled trials investigating the role of statins in patients with MASLD/MASH. Statins play a vital role in mitigating the cardiovascular risk associated with elevated plasma lipids, particularly in groups that have been shown to be at higher risk a priori. Beside the postulated effects on cardiovascular and liver endpoints, treatment with statins has been demonstrated to be safe, effective and their use is therefore strongly recommended in patients with MASLD and MASH with dyslipidemia.

Despite the apparent lack of data regarding the efficacy of other commonly prescribed lipid lowering agents (e.g.: of ezetimibe, bempedoic acid, PCSK-9 inhibitors) in the MASLD/MASH cohort, the addition of these should be considered if statin therapy fails to reach treatment goals [31]. 

### Incretin Mimetics

Since the release of the first glucagon-like peptide-1 (GLP-1) receptor agonist exenatide almost two decades ago, there has been an increased interest in the use of this medication class not only for the treatment of type 2 diabetes and obesity, but also to reduce risk in other cardiometabolic disease states such as MASLD and MASH. Liraglutide is a well-established GLP-1 receptor agonist for the treatment of type 2 diabetes and obesity [65]. In a small placebo-controlled phase II trial, treatment with liraglutide was associated with the resolution of non-alcoholic steatohepatitis with no signs of worsening fibrosis [66]. 

Another mono-directed GLP-1 agonist, semaglutide, also primarily developed for the treatment of type 2 diabetes, has shown promising results in reducing bodyweight in obese individuals with diabetes and resulted in a reduction of CVD events in patients with obesity but without diabetes [67–69]. Two phase II trials investigated the use of semaglutide in patients with MASH, with conflicting results [70, 71]. A currently enrolling phase 3 trial will help to fill the current gap in knowledge [72]. 

Tirzepatide, an agonist of the glucose-dependent insulinotropic polypeptide (GIP) and GLP-1 receptor, has shown promising weight-loss effects in patients with and without diabetes [73–75]. A recent, randomized, placebo-controlled phase 2 dose-finding trial in patients with MASH and moderate or severe fibrosis showed that treatment with tirzepatide for one year resulted in more effective MASH resolution without worsening of fibrosis as compared to placebo [76]. 

Survodutide, yet another GLP-1 agonist which additionally targets the glucagon receptor, has demonstrated dose-dependent reductions in body weight [77]. In patients with biopsy-confirmed MASH with fibrosis, one year treatment with survodutide was superior to placebo with respect to improvement in MASH without worsening of liver fibrosis [78]. 

As adequately powered phase 3 trials investigating various incretin agonists in MASLD and MASH with robust histological endpoints are still underway, current practice guidelines highlight the beneficial effects of treatment with GLP-1 agonists on non-liver related endpoints and for other comorbidities, such as diabetes or obesity, without generally endorsing this class of agents for the specific treatment of MASLD/MASH yet [31, 32]. However, recent data outlined above highlight a huge potential in the treatment of MASH showing resolution of steatohepatitis.

#### Sodium/Glucose Cotransporter-2 Inhibitors (SGLT-2 Inhibitors)

Several members of the SGLT-2 inhibitors class of medications have shown robust treatment effects in patients with type 2 diabetes mellitus as well as heart failure and chronic kidney disease and are therefore unequivocally recommended by clinical practice guidelines [79–81] for the treatment in aforementioned indications.

Several smaller trials have shown that commonly used SGLT2-inhibitors are able to lower liver fat content in patients with type 2 diabetes [82–84]. In a large, nationwide cohort study involving more than 80,000 patients with type 2 diabetes and MASLD in Korea, treatment with SGLT-2 inhibitors was associated with lower incidence rates of liver-related outcomes and regression [85] of MASLD. As there are currently no randomized controlled trials with histological liver endpoints available, current practice guidelines recommend use of SGLT-2 inhibitors in patients with MASLD within their approved indication [31, 32]. 

### Peroxisome Proliferator-Activated Receptor (PPAR)-Agonists

Various PPAR receptors agonists with effects on triglycerides and carbohydrate metabolism are available. Pioglitazone, a PPAR-γ agonist, improves histological features of liver fibroses. However, weight gain and fluid retention are among common side effects, calling for more randomized data for pioglitazone in this indication [86] before widespread use. Lanifibranor is a new, pan-PPAR agonist, which showed improvements in liver fibrosis and markers of cardiometabolic health in a phase IIb trial in patients with MASH [87, 88]. Treatment with another pan-PPAR agonist, elafibranor, did not demonstrate a statistically significant effect on resolution of MASH in a phase III trial. Overall, data on the role of PPAR-agonists in the treatment of MASLD/MASH is scarce with some potential benefits described [86, 87, 89]. 

### Bariatric Surgery

Despite the lack of prospective randomized studies in the context of CVD prevention and MASLD, retrospective studies and meta-analyses have suggested beneficial effects associated with bariatric surgery in both indications [90–92]. The most recent clinical practice guidelines recommend these procedures in otherwise healthy patients with a BMI ≥ 35 kg/m2 or in patients with a BMI ≥ 30 kg/m2 and type 2 diabetes, as well as patients with a BMI between 30 and 35 kg/m2 who did not achieve substantial or durable weight loss or improvement of comorbidities including MASLD using nonsurgical methods [93]. Although some caution in interpreting previous findings seems warranted, observational studies suggest significant cardiovascular risk reductions in patients with obesity and diabetes undergoing bariatric surgery [90, 94]. These findings are supported by recent meta-analyses which show favorable effects in all-cause mortality, CVD mortality and other cardiovascular outcomes [95, 96]. 

Beneficial effects of bariatric surgery were also seen in patients with MASLD and MASH. A recent meta-analysis including more than 2,000 patients with MASLD undergoing bariatric surgery suggested significant improvements of biochemical and histological markers of MASLD, with slowing of disease progression and even resolution of liver fibrosis in 30% of included patients [91]. Bariatric surgery can therefore be considered in patients fulfilling eligibility criteria, as the surgical intervention is considered safe and effective in this patient cohort. In patients with decompensated liver cirrhosis, metabolic surgery is associated with elevated levels of postoperative mortality and is therefore not routinely recommended [31]. 

## Conclusion

MASLD affects up to a third of the world population and is associated with a high risk for cardiovascular disease and associated adverse events. Therefore, such patients require a multi-modal treatment approach including regular cardiovascular risk assessments and optimization of cardiometabolic risk factors to prevent cardiovascular disease and adverse cardiovascular events. Such an approach should include, but not be limited to, regular physical activity, a healthy diet and weight loss as well as optimization of blood pressure, dysglycemia and hyperlipidemia. Statin therapy is safe and beneficial across the spectrum of MASLD and MASH and mildly elevated transaminases should not prevent providers to prescribe or continue with statin therapy in patients fulfilling clinical practice guideline recommendations. Novel approaches with evidence for lipid specific beneficial effects include the GLP-1 receptor agonist family. Data from larger phase III trials are therefore eagerly awaited.

## Key References

- Younossi ZM, Golabi P, Paik JM, Henry A, Van Dongen C, Henry L. The global epidemiology of nonalcoholic fatty liver disease (NAFLD) and nonalcoholic steatohepatitis (NASH): a systematic review. Hepatology. 2023 Apr;77(4):1335.
  - Findings from this review demonstrate the increasing global burden of MASLD and MASH.
- Targher G, Byrne CD, Tilg H. NAFLD and increased risk of cardiovascular disease: clinical associations, pathophysiological mechanisms and pharmacological implications. Gut. 2020 Sep;69(9):1691–705.
  - Finding from this review highlight the interplay between MASLD and cardiovascular disease.
- Rinella ME, Neuschwander-Tetri BA, Siddiqui MS, Abdelmalek MF, Caldwell S, Barb D, et al. AASLD Practice Guidance on the clinical assessment and management of nonalcoholic fatty liver disease. Hepatology. 2023 May;77(5):1797.
  - Current clinical practice guidelines on the management of nonalcoholic fatty liver disease issued by the American Association for the Study of Liver Diseases.

## Data Availability

No datasets were generated or analysed during the current study.
